# Non-uniform magnetic fields for collective behavior of self-assembled magnetic pillars

**DOI:** 10.1007/s11721-024-00240-z

**Published:** 2024-07-07

**Authors:** Juan J. Huaroto, Franco N. Piñan Basualdo, Dionne Lisa Roos Ariëns, Sarthak Misra

**Affiliations:** 1 Surgical Robotics Laboratory, Department of Biomechanical Engineering, University of Twente, 7522 NB Enschede, The Netherlands; 2 Surgical Robotics Laboratory, Department of Biomaterials and Biomedical Technology, University Medical Centre Groningen and University of Groningen, 9713 GZ Groningen, The Netherlands

**Keywords:** Collective behavior, Magnetic pillars, Electromagnetic actuation, Magnetic gradients

## Abstract

Programmable and self-assembled magnetic pillars are essential to expanding the application domain of magnetic microparticle collectives. Typically, the collective behavior of self-assembled magnetic pillars is carried out by generating uniform and time-varying magnetic fields. However, magnetic field-shaping capabilities employing non-uniform fields have not been explored for magnetic pillars. In this study, we generate non-uniform magnetic fields using a nine-coil electromagnetic system to achieve object manipulation, upstream/downstream locomotion, and independent actuation. We begin analyzing the static magnetic self-assembly of reduced iron microparticles and experimentally derive the average dimensions (height and diameter) of the resulting pillars. Subsequently, we delve into the collective dynamic response under non-uniform and time-varying magnetic fields, unveiling four distinct modalities. In order to demonstrate the versatility of our approach, we extend our study to the two-dimensional manipulation of a millimeter-sized glass bead using a precessing magnetic field describing a Lissajous curve. Moreover, we showcase the ability of magnetic pillars to adapt to confined and dynamic conditions within fluidic tubes. We finally present a noteworthy case where the nine-coil electromagnetic system independently actuates two clusters of magnetic pillars. Our study shows the potential of using non-uniform magnetic fields to actuate self-assembled magnetic pillars, enabling morphology reconfiguration capabilities, object manipulation, locomotion, and independent actuation.

**Supplementary Information:**

The online version contains supplementary material available at 10.1007/s11721-024-00240-z.

## Introduction

Collective behavior is a widespread phenomenon observed in nature across all scales (Whitesides & Grzybowski, 2002; Vicsek & Zafeiris, 2012), where groups of individual agents exhibit different coordinated/collective actions (Okubo, 1986). This phenomenon has been studied extensively in fields such as biology (Deneubourg et al., 1986) and physics (Mori, 1965). The existence of coordinated behavior can lead to emergent properties not present in the individual agents themselves (Liljeström et al., 2019), which has inspired the development of multiple robotic systems (Rubenstein et al., 2012; Werfel et al., 2014). These collective properties can be harnessed to accomplish complex tasks and adapt to dynamic environments robustly (Gardi et al., 2022).

In microrobotics, collective systems are advantageous over individual micro-agents since the latter are limited to performing specific tasks (Elgeti et al., 2015). Collective systems can be reorganized in situ and accomplish complex actions such as manipulation and cargo transport (Ahmed et al., 2021; Yang et al., 2022; Xu & Xu, 2022). Thus, understanding the interaction and self-organization of micro-agents is fundamental to developing novel collaborative applications. Furthermore, contactless actuation methods driven by magnetic fields (Yang et al., 2021; Xu et al., 2023), acoustic waves (Zhou et al., 2016; Liu et al., 2022), and light (Hu et al., 2022) can be potentially implemented under in vitro and ex vivo conditions, which enables their use in biomedical scenarios.

Magnetic actuation is a promising method for controlling collective systems due to its transparency and biocompatibility (Sitti & Wiersma, 2020). Magnetic collective systems can perform multimodal locomotion (Xie et al., 2019), morphology reconfiguration (Dong & Sitti, 2020), object/droplet manipulation (Kim et al., 2015; Sun et al., 2021), and cargo transportation (Akter et al., 2022). Inspired from cilia structures in nature, recent studies have proposed magnetic micropillars for generating programmable metachronal waves (Dong et al., 2020), enhanced object manipulation (Demirörs et al., 2021), and liquid/solid transport (Miao et al., 2022; Sohn et al., 2023). Magnetic micropillars are primarily fabricated using replica molding (Ren et al., 2022; Cui et al., 2023) and self-assembly of magnetic soft composites (i.e., a blend of silicone and magnetic microparticles) (Miao et al., 2022; Demirörs et al., 2021). Mobile permanent magnets or static arrangements of electromagnetic coils have been used to actuate magnetic micropillar arrays (Ni & Wang, 2023). Although the aforementioned fabrication methods permitted the engineering of the magnetization direction of micropillars, the pillars were attached to a substrate, restricting the morphology reconfiguration of pillars (Sohn et al., 2023). Finding a strategy to fabricate programmable pillars by magnetic self-assembly techniques is a current challenge in the development process (Ni & Wang, 2023).

Self-assembly is the spontaneous organization of individual agents into a stable configuration. In particular, magnetic self-assembly has been applied to control the collective behavior of magnetic droplets (Timonen et al., 2013; Wang et al., 2018), soft ferromagnetic beads (Collard et al., 2022), and generate programmable pillars (Xu & Xu, 2022). Self-assembled magnetic pillars use uniform magnetic fields for actuation, demonstrating efficient deployment on biological tissue, navigation through uneven surfaces, and cargo transportation (Xu & Xu, 2022; Xu et al., 2023). However, the homogeneous characteristics of the magnetic field can limit the field-shaping capabilities and generation of gradients. Prior work in electromagnetic actuation exploits non-uniform magnetic fields generated by arrangements of multiple electromagnets for independent manipulation of microparticles (Diller et al., 2013; Ongaro et al., 2018b; Piñan Basualdo & Misra, 2023). Therefore, expanding the application domain of non-uniform magnetic fields to magnetic self-assembled magnetic pillars has the potential to achieve morphology reconfiguration and independent actuation of self-assembled magnetic pillar clusters.

To generate programmable and fabrication-free pillars based on the self-assembly of magnetic microparticles, we exploit the field-shaping capabilities of a nine-coil electromagnetic system (Ongaro et al., 2018b). This electromagnetic system can generate time-varying non-uniform magnetic fields to explore novel actuation modalities, which are used for object manipulation and locomotion within fluidic tubes. In order to show the potential of non-uniform magnetic fields, we exploit the spatially selective actuation of the electromagnetic system to perform independent actuation of magnetic pillar collectives. We start analyzing the static magnetic self-assembly of the microparticles by experimentally obtaining the average dimensions of the magnetic pillars. Additionally, we study the pillars’ collective dynamic response to non-uniform and time-varying magnetic fields, identifying four primary modalities. Furthermore, we demonstrate that a precessing magnetic field describing a Lissajous curve (i.e., parametric curve composed of two perpendicular sinusoidal signals with different phases and frequencies) can be utilized for the two-dimensional manipulation of a millimeter-sized glass bead. We study the behavior of the pillars in confined and dynamic environments by actuating them within fluidic tubes. We showcase the capability of the nine-coil electromagnetic system by performing independent actuation of two clusters of magnetic pillars. Our results show that non-uniform magnetic fields are a promising approach for actuating self-assembled magnetic pillars, enabling reconfiguration capabilities, object manipulation, locomotion, and independent actuation.

## Results and discussion

**Fig. 1:**
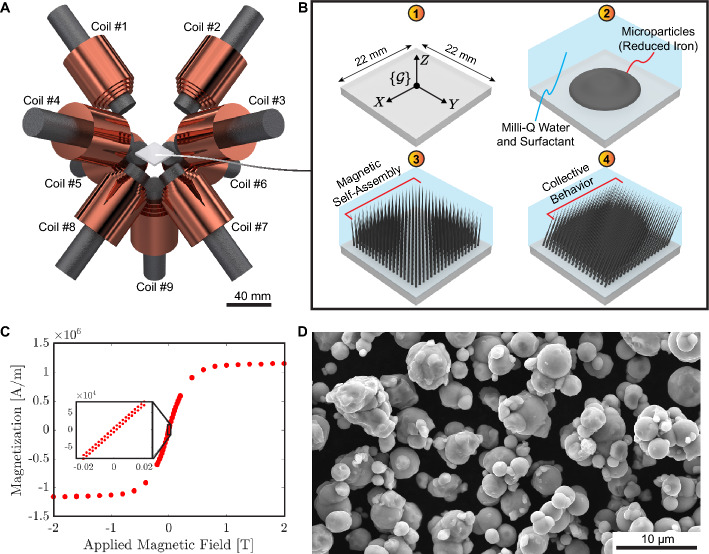
Self-assembled magnetic pillars generated from microparticles of reduced iron. **A** Nine-coil electromagnetic actuation system. **B** Generation of self-assembled magnetic pillars and collective behavior. The states of the microparticles of reduced iron are indicated as follows: $\textcircled {1}$, base of the workspace surrounded the electromagnetic actuation system; $\textcircled {2}$, magnetic microparticles of reduced iron located within the workspace along with Milli-Q water and surfactant; $\textcircled {3}$, the coil #9 is activated with a constant current to generate the self-assembled magnetic pillars; $\textcircled {4}$, the coils #1–8 are activated to generate non-uniform and time-varying magnetic fields, which produce the collective behavior of magnetic pillars. **C** Magnetization curve of reduced iron. **D** Scanning electron microscopy (SEM) of microparticles of reduced iron

The generation of magnetic self-assembly and collective behavior is carried out in a nine-coil electromagnetic system (Fig. 1A). Microparticles of reduced iron are placed into a polymethyl methacrylate (PMMA) box (base dimensions of 22 $\times$ 22 mm$^2$) along with pure water (i.e., Milli-Q water) and surfactant (details in Sect. 4). The PMMA box containing the microparticles and suspension is located within the electromagnetic system workspace. A constant current is used to power the coil #9 as it generates the magnetic self-assembly of microparticles (i.e., magnetic pillars). Thereon, the coils #1–8 generate non-uniform time-varying magnetic fields and gradients, which trigger the collective behavior of magnetic pillars (Fig. 1B). The magnetization curve of reduced iron in Fig. 1C shows negligible remanence (magnetically soft material) and a linear behavior if the applied magnetic field is below 250 mT. The morphology of the microparticles is acquired from scanning electron microscopy, which shows particles with non-uniform diameters (1–5 $\upmu$m) (Fig. 1D). In order to study the collective behavior of magnetic pillars, we divide our analysis into five sections: (1) static self-assembly of microparticles of reduced iron, (2) collective dynamic behavior of magnetic pillars, (3) object manipulation, (4) upstream and downstream locomotion, and (5) independent actuation of two clusters of magnetic pillars.

### Static magnetic self-assembly

**Fig. 2:**
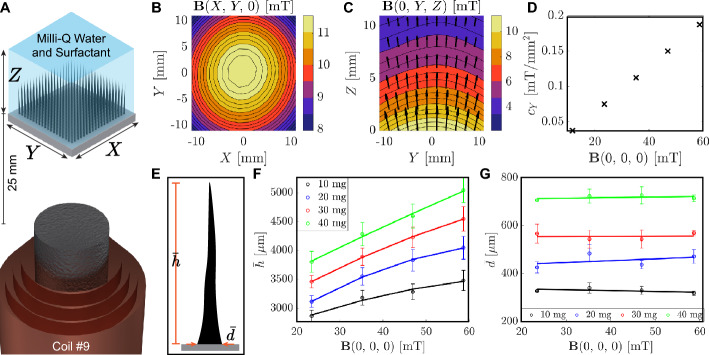
Characterization of static magnetic self-assembly of microparticles of reduced iron. **A** Workspace configuration with respect to coil #9. Magnetic field maps in the **B** XY and **C** YZ planes (powering coil #9 with 1 A). **D** Confining curvature of the magnetic field ($c_Y$) as a function of the magnetic field at the center of the workspace $\left( \textbf{B}{(0,0,0)}\right)$. **E** Illustration of a magnetic pillar and average geometric parameters ($\bar{h}$ and $\bar{d}$). Experimental characterization of **F** the average height ($\bar{h}$) and **G** base diameter ($\bar{d}$) varying $\textbf{B}{(0,0,0)}$ and mass of microparticles of reduced iron

We begin the study by analyzing the self-assembly of microparticles of reduced iron to create magnetic pillars. The microparticles are magnetized by an external magnetic field generated by coil #9, as depicted in Fig. 2A. Using the current-to-field map of the system, the magnetic field generated by powering coil #9 with 1 A is shown for the XY-/YZ-planes, with black arrows representing the magnetic field direction (Fig. 2B, C). In order to assess the uniformity of the magnetic field, we compute the curvature of the magnetic field at the center of the workspace. Unlike previous studies, which use permanent magnets at varying distances to modify the curvature (Xu & Xu, 2022; Ni & Wang, 2023), we investigate the capabilities of an iron-cored electromagnet to create magnetic pillars while maintaining a constant distance from the workspace (25 mm). Considering the magnetic field is cylindrical symmetric along the coil axis, the curvature along the X and Y-axis exhibit identical values for a static magnetic field. We compute the curvature along the Y-axis $\left( c_Y = -\frac{\text {d}^2|\textbf{B}(0,0,0)|}{\text {d}Y^2}\right)$ as a function of the magnetic field $\left( \textbf{B}(0,0,0)\right)$. Our results demonstrate that the curvature ($c_Y$) varies linearly with the current of coil #9 and the magnitude of $\textbf{B}(0,0,0)$ (Fig. 2D).

By varying the current of coil # 9 ($I_9$ = 2–5 A) and mass of microparticles of reduced iron ($m_{ri}$ = 10–40 mg), we estimate the magnetic field at the center of the workspace ($\textbf{B}(0,0,0)$) and compute the average height ($\bar{h}$) and base diameter ($\bar{d}$) of magnetic pillars (Fig. 2E). The cameras located at the side and above the workspace permit the acquisition of images, which are subsequently processed to compute $\bar{h}$ and $\bar{d}$, respectively (S1 Text, Supplementary information). Figure 2F, G shows that $\bar{h}$ increases according to the magnetic field while $\bar{d}$ remains approximately constant. On the other hand, $\bar{h}$ and $\bar{d}$ show and increment according to the mass of reduced iron microparticles within the workspace. It is worth noting that the increment of current through coil #9 is realized abruptly, and the coil is powered off ($I_9$ = 0 A) between measurements. This way, we reduce the influence between adjacent experiments.

### Dynamic collective behavior

**Fig. 3:**
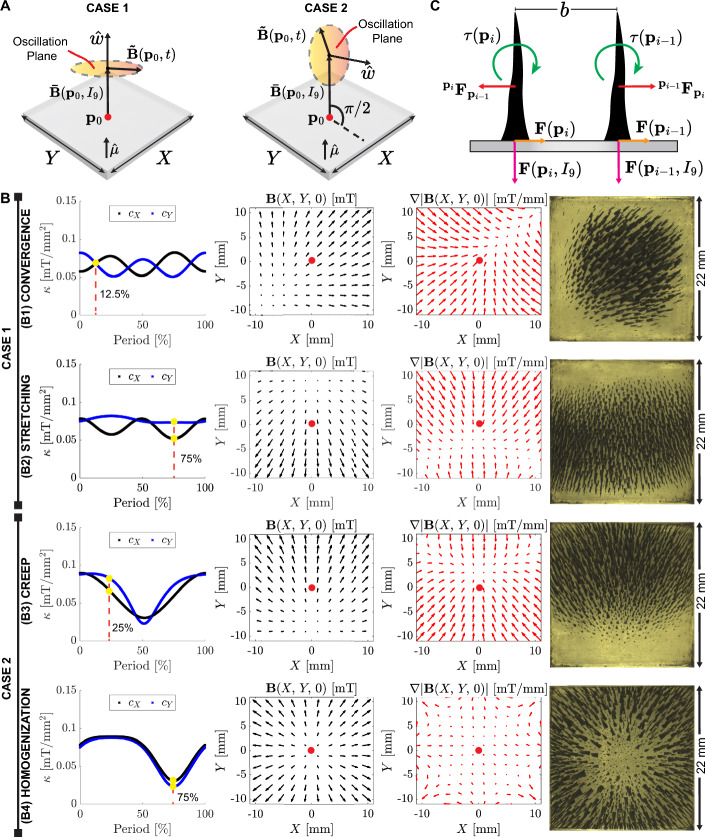
Collective behavior of magnetic pillars. **A** Two cases of actuation modalities according to the spatial configuration of unitary vectors (${\varvec{\hat{\mu }}}$ and ${\varvec{\hat{\omega }}}$) perpendicular to the base of the workspace and oscillation plane, respectively. The point ($\textbf{p}_0 = [0,\,0,\,0]^T$) is defined to illustrate the components of the magnetic field $\left( \varvec{\bar{B}}(\textbf{p}_0,I_9)\,\text {and}\,\varvec{\tilde{B}}(\textbf{p}_0,t)\right)$. **B** Four actuation modalities are proposed to generate different collective behavior of magnetic pillars. The non-uniformity of the magnetic field is analyzed using directional curvatures $\left( \kappa = [c_X,\,c_Y]\right)$ at $\textbf{p}_0$. In addition, the magnetic field $\left( \textbf{B}(X,Y,0)\right)$, gradients $\left( \nabla |\textbf{B}(X,Y,0)|\right)$, and an image frame of the collective behavior of magnetic pillars are provided. The red dots indicate the center of the workspace ($\textbf{p}_0$). *Please refer to the accompanying video* (S1 Video, Supplementary information). **C** Illustration of the external $\left( \textbf{F}(\textbf{p}_i),\,\textbf{F}(\textbf{p}_{i-1}),\,\textbf{F}(\textbf{p}_i,I_9),\,\textbf{F}(\textbf{p}_{i-1},I_9)\right)$, internal forces ($^{\textbf{p}_{i-1}}\textbf{F}_{\textbf{p}_i}\,\text {and}\,^{\textbf{p}_i}\textbf{F}_{\textbf{p}_{i-1}}$), and torques $\left( {\varvec{\tau }}(\textbf{p}_i)\,\text {and}\,{\varvec{\tau }}(\textbf{p}_{i-1})\right)$ applied to adjacent magnetic pillars (Color figure online)

The study of magnetic self-assembly of microparticles of reduced iron provides valuable insights into the formation of magnetic pillars under non-uniform and static magnetic fields. In this section, we focus on exploring the collective behavior of these magnetic pillars when exposed to non-uniform and time-varying magnetic fields. To this end, we represent all magnetic fields at the center of the workspace ($\textbf{p}_0 = [0,\,0,\,0]^{T}$) (Fig. 3A). The magnetic pillars are generated from the static magnetic field created by coil #9 ($\varvec{\bar{B}}(\textbf{p}_0, I_9)$) (Fig. 3A) while coils #1–8 are utilized to create time ($t \in \mathbb {R}^+$) varying magnetic fields ($\varvec{\tilde{B}}(\textbf{p}_0,t)$). By considering the spatial configuration of unitary vectors (${\varvec{\hat{\mu }}}$ and ${\varvec{\hat{\omega }}}$), which are perpendicular to the base of the workspace and the oscillation plane, respectively. Prior research has explored precessing and oscillating fields to perform multimodal locomotion of self-assembled magnetic pillars (Yigit et al., 2019; Xu & Xu, 2022; Xu et al., 2023). Drawing inspiration from the previous body of literature, we define two distinct cases for the actuation modalities (Fig. 3A). The resulting magnetic field at the center of the workspace ($\textbf{B}(\textbf{p}_0)$) for cases 1 (${\varvec{\hat{\omega }}} \parallel {\varvec{\hat{\mu }}}$) and 2 (${\varvec{\hat{\omega }}} \perp {\varvec{\hat{\mu }}}$) are mathematically defined as follows:

1 $$\begin{aligned} {\left\{ \begin{array}{ll} \text {Case 1: }\textbf{B}(\textbf{p}_0) = \varvec{\bar{B}}(\textbf{p}_0,I_9) + \varvec{\tilde{B}}_X(\textbf{p}_0,t) + \varvec{\tilde{B}}_Y(\textbf{p}_0,t)\\ \text {Case 2: }\textbf{B}(\textbf{p}_0) = \varvec{\bar{B}}(\textbf{p}_0,I_9) + \varvec{\tilde{B}}_{X}(\textbf{p}_0,t) + \varvec{\tilde{B}}_Y(\textbf{p}_0,t) + \varvec{\tilde{B}}_Z(\textbf{p}_0,t), \end{array}\right. } \end{aligned}$$

where the magnetic fields $\left( \varvec{\tilde{B}}_X(\textbf{p}_0,t),\,\varvec{\tilde{B}}_Y(\textbf{p}_0,t),\, \text {and}\,\varvec{\tilde{B}}_Z(\textbf{p}_0,t)\right)$ are the components of $\varvec{\tilde{B}}(\textbf{p}_0,t)$, and are represented by sinusoidal functions with amplitudes ($A_X$, $A_Y$, $A_Z$), frequencies ($f_X$, $f_Y$, $f_Z$), and phases ($\varphi _X$, $\varphi _Y$, $\varphi _Z$), respectively. According to the aforementioned cases and the observed collective behavior generated by the magnetic pillars, we propose the following actuation modalities: convergence, stretching, creep, and homogenization. The actuation modalities are experimentally tested using 30 mg of microparticles of reduced iron and using the parameters presented in Table 1 (Fig. 3B). For providing a metric on the non-uniformity of the magnetic field, each modality is analyzed through the directional curvatures of the magnetic field at the center of the workspace $\left( \kappa = \left[ c_X = -\frac{\text {d}^2|\mathbf {B_{(0,0,0)}}|}{\text {d}X^2},\,c_Y = -\frac{\text {d}^2|\mathbf {B_{(0,0,0)}}|}{\text {d}Y^2}\right] \right)$. Moreover, the magnetic field $\left( \textbf{B}{(X, Y, 0)}\right)$, gradient $\left( \nabla |\textbf{B}{(X, Y, 0)}|\right)$, and image frame are shown at a specific percentage of the oscillation period (yellow points) (Fig. 3B).

Prior to discussing each actuation modality, we provide an illustration that depicts all the forces and torques acting on two adjacent pillars (identified by the subscripts $i$ and $i-1$) exposed to a non-uniform magnetic field. We assume that each magnetic pillar is composed of ($N \in \mathbb {N}$) microparticles of reduced iron. By powering the electromagnetic coil #9 with a constant current, we have observed the generation of magnetic pillars within the workspace. Each pillar behaves as a magnetic dipole since the microparticles of reduced iron are magnetized in response to the external magnetic field. The magnetic gradients generated by powering the coil #9 create forces ($\textbf{F}(\textbf{p}_i, I_9)$ and $\textbf{F}(\textbf{p}_{i-1}, I_9)$) that are oriented toward the base of the workspace to maintain the shape and structural integrity of the magnetic pillars. Furthermore, each pillar interacts with its adjacent pillar through a repulsive force ($^{\textbf{p}_{i-1}}\textbf{F}{\textbf{p}_i} = -\,^{\textbf{p}_i}\textbf{F}{\textbf{p}_{i-1}}$), which keeps the pillars separated by a distance ($b \in \mathbb {R}^{+}$) (Fig. 3C). Using coils #1–8, we generate non-uniform and time-varying magnetic fields and gradients. These magnetic fields and gradients exert torques $\left( {\varvec{\tau }}(\textbf{p}_i)\right)$ and $\left( {\varvec{\tau }}(\textbf{p}_{i-1})\right)$ as well as forces $\left( \textbf{F}(\textbf{p}_i)\right)$ and $\left( \textbf{F}(\textbf{p}_{i-1})\right)$ on the magnetic pillars, making them susceptible to tilting and slipping, respectively (Fig. 3C). It is important to note that, for the purpose of analysis, we are neglecting friction and viscous forces.

By understanding the influence of the magnetic field and gradients, we can gain further insights into the collective behavior of magnetic pillars under non-uniform and time-varying magnetic fields. We begin by discussing the actuation modalities corresponding to case 1. The magnetic field oscillates in a parallel plane with respect to the workspace. Thus, the forces ($\textbf{F}(\textbf{p}_i, I_9)$) remain constant during a complete period (Fig. 3C). Moreover, the torques (${\varvec{\tau }}(\textbf{p}_i)$) and forces ($\textbf{F}(\textbf{p}_i)$) determine the motion of the magnetic pillar based on the time-varying magnetic fields and gradients, respectively. In particular, the convergence modality exhibits a rotating magnetic field, causing the curvatures along the X and Y-axes to oscillate reciprocally (Fig. 3B1). The magnetic pillars align themselves according to the magnetic field (black arrows), while the gradients (red arrows) generate forces, which tend to gather the magnetic pillars toward the center of the workspace, forming a circumference (S1 Video: 00:07–00:34 s, Supplementary information). The circularity of this circumference can be controlled by adjusting the values of the magnetic field amplitudes ($A_X$ and $A_Y$). On the other hand, the stretching modality can be considered a sub-case of the convergence modality, where the magnetic field along the X-axis is set to zero ($A_X = 0$ mT). As a consequence, the collective of magnetic pillars tends to stretch along the X-axis over time (S1 Video: 00:35–01:01 s, Supplementary information). Typically, the collective can be stretched along different axes by adjusting the values of $A_X$ and $A_Y$.

For actuation modalities corresponding to case 2, the effective magnetic field along the Z-axis varies according to the amplitude of the oscillating magnetic field along the Z-axis ($A_Z$). Therefore, we impose the following ratio to maintain the magnetic self-assembly of microparticles of reduced iron: $\frac{A_Z}{|\varvec{\bar{B}}(\textbf{p}_0,I_9)|} < 1$. In our experiments, we utilize a maximum ratio ($\approx 0.34$) such that the magnitude of the static magnetic field predominates over the oscillating one. Since the effective magnetic field along the Z-axis oscillates over time. The curvatures along the X and Y-axes reach a minimum value when $\varvec{\bar{B}}(\textbf{p}_0,I_9)$ and $\varvec{\tilde{B}}(\textbf{p}_0,t)$ have opposite directions. In particular, the creep modality uses a rotating magnetic field with components along the Y and Z-axes ($A_Y$ and $A_Z$) (Fig. 3B3). This way, the magnetic pillars are displaced in response to the resultant magnetic field. (S1 Video, Supplementary information: 01:02–01:26 s). The predefined maximum ratio ($\approx 0.34$) prevents the magnetic pillars from rotating as their base remains in contact with the base of the workspace. Furthermore, the creep orientation can be modified by introducing a component along the X-axis ($A_X$) with the same frequency and phase as the one along the Y-axis ($f_X = f_Y$ and $\varphi _X = \varphi _Y$). This actuation modality is subsequently used for experiments involving the motion of magnetic pillars in fluidic tubes. The homogenization modality is a particular case of the creep modality, generating only an oscillating magnetic field along the Z-axis. The simulation and experimental results show that the magnetic pillars tend to move toward the boundaries and the center of the workspace (S1 Video, Supplementary information: 01:27–01:56 s s). This collective behavior leads to a reorganization (i.e., homogenization) of magnetic pillars within the workspace. We utilize the homogenization modality to reorganize the magnetic pillars between experiments.

**Table 1:** Actuation modalities for self-assembled magnetic pillars

Modality	$I_9$ [A]	$A_X$ [mT]	$A_Y$ [mT]	$A_Z$ [mT]	$f_X$ [Hz]	$f_Y$ [Hz]	$f_Z$ [Hz]	$\varphi _X$ [rad]	$\varphi _Y$ [rad]	$\varphi _Z$ [rad]
Convergence	2	10	10	0	1	1	0	0	$\pi /2$	0
Stretching	2	0	10	0	0	1	0	0	0	0
Creep	2	0	8	8	0	1	1	0	0	$\pi /2$
Homogenization	2	0	0	8	0	0	1	0	0	0

### Object manipulation

**Fig. 4:**
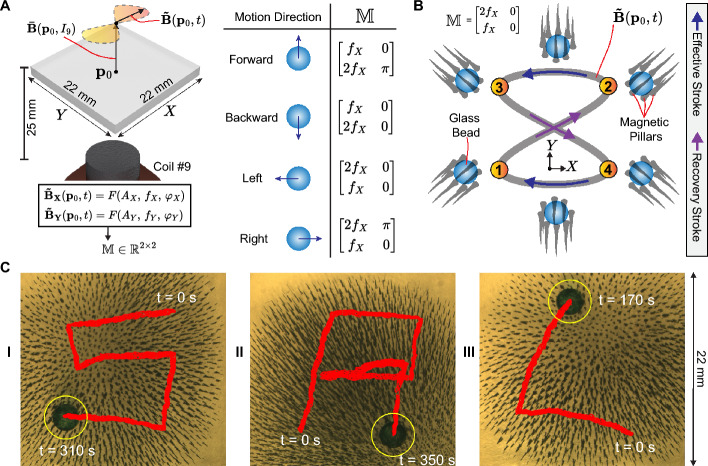
Manipulation of a passive object (2 mm glass bead). **A** The components of the magnetic field: Constant magnetic field generated by the coil #9 $\left(\varvec{\bar{B}}(\textbf{p}_0, I_9)\right)$, and oscillating magnetic field $\left( \varvec{\tilde{B}}(\textbf{p}_0,t)\right)$ with amplitudes $A_X$ and $A_Y$ along the X and Y-axis, respectively. The matrix ($\mathbb {M} \in \mathbb {R}^{2 \times 2}$), which encompasses the frequencies ($f_X$, $f_Y$) and phases ($\varphi _X$, $\varphi _Y$), is used to encode the direction of motion of the bead. **B** Illustration of the periodic motion performed by the magnetic pillars to move the 2 mm glass bead towards the left. The precessing magnetic field $\left( \varvec{\tilde{B}}(\textbf{p}_0,t) + \varvec{\bar{B}}(\textbf{p}_0, I_9)\right)$ describes a Lissajous curve according to the corresponding matrix $\mathbb {M}$. The effective stroke is achieved within the intervals $\textcircled {2}$ $\rightarrow$ $\textcircled {3}$ and $\textcircled {4}$ $\rightarrow$ $\textcircled {1}$, whereas the recovery stroke is performed within $\textcircled {1}$ $\rightarrow$ $\textcircled {2}$ and $\textcircled {3}$ $\rightarrow$ $\textcircled {4}$. **C** Manipulation of a 2 mm glass bead (encircled in yellow). The images show the trajectory (in red) of the glass bead as it describes the alphabet I: “S,” II: “R,” and III: “L,” respectively. The insets show the initial and final time stamps. *Please refer to the accompanying video* (S2 Video, Supplementary information) (Color figure online)

The dynamic collective behavior of magnetic pillars, achieved through the self-assembly of iron microparticles, presents a concept primarily explored for the assembly/disassembly of various spherical particles (diameter $\le$ 1 mm and mass < 1 mg) (Xu & Xu, 2022). However, the challenge remains in achieving two-dimensional and dexterous manipulation of millimeter-sized objects while simultaneously preserving the positional integrity of the collective of magnetic pillars. In this study, we conduct the manipulation of a 2 mm glass bead (mass of 9.3 mg) using magnetic pillars within a workspace containing Milli-Q water and surfactant. The microparticles of reduced iron (20 mg mass) and the bead are positioned within the workspace while powering coil #9 $\left(\varvec{\bar{B}}(\textbf{p}_0, I_9)\right)$ to generate the magnetic pillars. Next, we employ a time-varying magnetic field $\left(\varvec{\tilde{B}}(\textbf{p}_0,t)\right)$ utilizing coils #1–8 to orient the magnetic pillars. The resulting precessing magnetic field is illustrated in Fig. 4A and mathematically represented as follows:

2 $$\begin{aligned} \textbf{B}(\textbf{p}_0) = |\varvec{\bar{B}}(\textbf{p}_0,I_9)|{\varvec{\hat{k}}} +10\sin {(2\pi f_X t + \varphi _X)}{\varvec{\hat{i}}} + 10\sin {(2\pi f_Y t + \varphi _Y)}{\varvec{\hat{j}}}\,\text {[mT]}, \end{aligned}$$

where $I_9=2\,\text{A},|\varvec{\bar{B}}(\textbf{p}_0,I_9)|=21.1\,{\text{mT}},$ and the unitary vectors (${\varvec{\hat{i}}}$, ${\varvec{\hat{j}}}$ and ${\varvec{\hat{k}}}$) corresponds to the X-/Y-/Z-axis, respectively. The precessing magnetic field $\left(\varvec{\tilde{B}}(\textbf{p}_0,t) + \varvec{\bar{B}}(\textbf{p}_0, I_9)\right)$ describes a Lissajous curve depending on the parameters of frequency ($f_X$ and $f_Y$) and phase ($\varphi _X$ and $\varphi _Y$) along the X-/Y-axis. We arrange these parameters as components of a matrix $$\left( \mathbb {M} = \begin{bmatrix}f_X & \varphi _X \\ f_Y & \varphi _Y \end{bmatrix}\right)$$, which is used to encode the following fundamental motion directions: forward, backward, left, and right (Fig. 4A). Figure 4B depicts the Lissajous curve used to move the bead toward the left direction $$\left( \mathbb {M} = \begin{bmatrix}2\,f_X & 0 \\ f_X & 0 \end{bmatrix}\right)$$. For the analysis, we divide the Lissajous curve into four segments (Fig. 4B). We experimentally demonstrate that the Lissajous curve permits the relative displacement of the glass bead with respect to the magnetic pillars collective (details in S1 Text, Supplementary information). The intervals $\textcircled {4}$ $\rightarrow$ $\textcircled {1}$ and $\textcircled {2}$ $\rightarrow$ $\textcircled {3}$ allow for the effective stroke of the bead. Previous research using precessing magnetic fields demonstrates that the Lissajous curve can generate non-reciprocal motion of soft magnetic microparticles (Collard et al., 2020). Based on the experimental results depicted in Figure S2 (S1 Text, Supplementary information), we demonstrate that the trajectory described by the glass bead can be decomposed into translation and a cyclic motion following the input Lissajous curve. During an actuation cycle, the bead follows the pillars’ motion. However, at the end of the cycle, the bead does not return to its initial position, exhibiting a net translation. Since this displacement is always in the same direction relative to the input Lissajous curve, it suffices to change the input signal orientation to select the advancement direction. In the object manipulation experiments, the four motion directions are tested to replicate trajectories that depict the alphabet “S,” “R,” and “L” (representing the initials of Surgical Robotics Laboratory) (Fig. 4C).

### Upstream and downstream locomotion

The capability of magnetic pillars to withstand and move within hollow cavities transporting fluid holds importance for studying potential biomedical applications under ex vivo conditions. We start testing the capability of the magnetic pillars to withstand fluid flow. To this end, we place 10 mg of microparticles of reduced iron inside a glass tube (8 mm inner diameter), connected by one of its ends to a syringe pump. The magnetic pillars are formed by circulating a constant current through coil #9 ($I_9$ = 2 A). Milli-Q water is automatically pumped at the maximum flow rate capacity (100 ml/min) to evaluate the displacement of the magnetic pillars (S3 Video, Supplementary information: 00:07–00:40 s). Our results show that the center of mass of magnetic pillars moves approximately 1.4 mm (for a duration of 15 s). Despite the susceptibility of the magnetic pillars to be carried away by the fluid flow, we observe that the collective of pillars withstands the drag forces acting upon it. Furthermore, experiments with 80 ml/min and 60 ml/min show a displacement of less than 0.5 mm for a duration of 15 s.

**Fig. 5:**
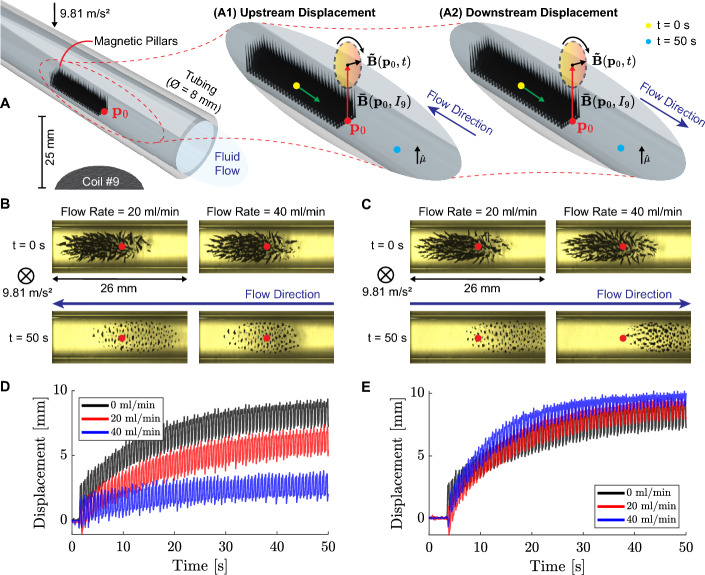
Motion of magnetic pillars within a tube transporting Milli-Q water. **A** Magnetic pillars formed from 10 mg of microparticles of reduced iron exposed to a constant magnetic field ($|\varvec{\bar{B}}(\textbf{p}_0,I_9)| = 21.1$ mT, with $I_9$ = 2 A). The creep modality, which employs a rotating magnetic field ($\varvec{\tilde{B}}(\textbf{p}_0,t)$), is utilized to move the pillars upstream (A1) and downstream (A2) the fluid flow. The yellow and pink dots represent the starting and final point, respectively (for a duration of 50 s). Top views of the starting and final frames of magnetic pillars displacing **B** upstream and **C** downstream using flow rates of 20 ml/min and 40 ml/min. Displacement of the center of mass of magnetic pillars in **D** upstream and **E** downstream conditions. The frames corresponding to the times t = 0 s and 50 s show the magnetic pillar states when the electromagnetic system is deactivated and activated, respectively. *Please refer to the accompanying video* (S3 Video, Supplementary information) (Color figure online)

Following our study, we test the locomotion of magnetic pillars upstream and downstream using the creep modality ($f_Y$ = $f_Z$ = 1.5 Hz and $A_Y$ = $A_Z$ = 8 mT) within a fluidic tube (Fig. 5A). The experiments are carried out for a duration of 50 s (Fig. 5B, C). The center of mass of the magnetic pillars is tracked while performing periodic motion according to the actuation frequency. The black lines depicted in Fig. 5D, E represent the displacement in still water. As the flow rate increases (20 ml/min and 40 ml/min), we observe that the upstream displacement of the center of mass decreases by 47.3% and 64.0%, respectively (Fig. 5D). Conversely, for downstream experiments, the center of mass displacement increases with the flow rate by 13.3% and 29.0%, respectively (Fig. 5E). Our results demonstrate that the magnetic pillars generated using 10 mg of reduced iron can navigate upstream and downstream, utilizing a maximum flow rate of 40 ml/min. Furthermore, the magnetic pillars can structurally withstand a fluid flow of 100 ml/min. It is worth noting that the saturation tendency on the displacement observed in Fig. 5D, E is due to the gradient generated by coil #9, which attracts the magnetic pillars toward the center of the workspace. In order to reach a more significant displacement, the electromagnetic coil arrangement can be assembled at the end effector of robotic platforms to constantly relocate the magnetic pillars collective (Yang et al., 2019; Wang et al., 2021).

### Independent actuation of two clusters of magnetic pillars

**Fig. 6:**
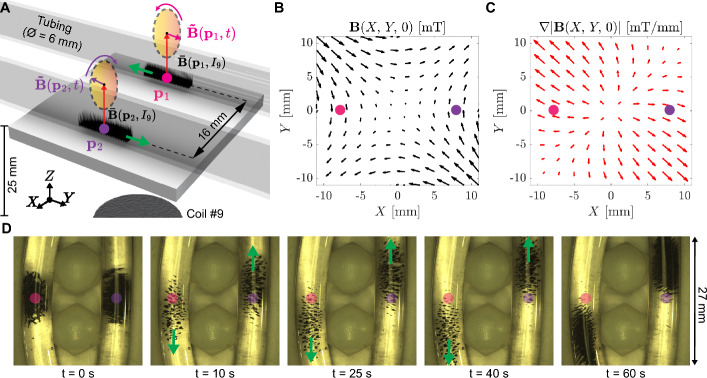
**A** Illustration of the independent actuation of two clusters of magnetic pillars. The magnetic pillars are formed from 10 mg of microparticles of reduced iron exposed to a constant magnetic field ($|\varvec{\bar{B}}(\textbf{p}_0,I_9)| = 21.1$ mT, with $I_9$ = 2 A). Two points ($\textbf{p}_1$ and $\textbf{p}_2$) within the workspace are used to generate two different oscillating magnetic fields $\left( \varvec{\tilde{B}}(\textbf{p}_1,t)\,\text {and}\,\varvec{\tilde{B}}(\textbf{p}_2,t)\right)$. **B** and **C** show the magnetic field and gradient within the workspace at t = 0.25 s (25 % of one actuation period), respectively. The pink and purple dots represent the points $\textbf{p}_1$ and $\textbf{p}_2$, respectively. **D** Time frames show the independent motion of two clusters of magnetic pillars. *Please refer to the accompanying video* (S4 Video, Supplementary information) (Color figure online)

The previous experiments are carried out using non-uniform and time-varying magnetic fields, which are the result of imposing a magnetic field at the center of the workspace ($\textbf{p}_0 = [0,\,0,\,0]^T$). Here, we leverage the capabilities of nine iron-cored coils to perform the independent motion of two clusters of magnetic pillars. To this end, we impose two rotating magnetic fields ($\varvec{\tilde{B}}(\textbf{p}_1,t)$ and $\varvec{\tilde{B}}(\textbf{p}_2,t)$) defined at two points ($\textbf{p}_1 = [-8,\,0,\,0]^T$ mm and $\textbf{p}_2 = [8,\,0,\,0]^T$ mm) within the workspace (Fig. 6A). The magnetic fields exhibit opposite directions, characterized by amplitudes of 8 mT and frequencies of 1 Hz. The resultant magnetic fields $\left( \textbf{B}{(\textbf{p}_1})\,\text {and}\,\textbf{B}{(\textbf{p}_2})\right)$ are defined as follows:

3 $$\begin{aligned} {\left\{ \begin{array}{ll} \textbf{B}{(\textbf{p}_1}) = |\varvec{\bar{B}}(\textbf{p}_1,I_9)|{\varvec{\hat{k}}} - 8\sin {(2\pi t )}{\varvec{\hat{j}}} + 8\cos {(2\pi t)}{\varvec{\hat{k}}}\,\text {[mT]}\\ \textbf{B}{(\textbf{p}_2}) = |\varvec{\bar{B}}(\textbf{p}_2,I_9)|{\varvec{\hat{k}}} + 8\sin {(2\pi t )}{\varvec{\hat{j}}} + 8\cos {(2\pi t)}{\varvec{\hat{k}}}\,\text {[mT]}, \end{array}\right. } \end{aligned}$$

where ${\varvec{\hat{i}}}$, ${\varvec{\hat{j}}}$ and ${\varvec{\hat{k}}}$ are the unitary vectors along the X-/Y-/Z-axis, respectively. Moreover, $|\varvec{\bar{B}}(\textbf{p}_1, I_9)| \approx |\varvec{\bar{B}}(\textbf{p}_2, I_9)| = 19.3\,\text{mT (using}\,I_9 = 2\,\text{A)}$, since the magnetic field generated by the coil #9 is cylindrical symmetric along the coil axis (Fig. 2B, C). Using the current-to-field map of the system, we obtain the magnetic field and gradients, where the pink and purple points represent $\textbf{p}_1$ and $\textbf{p}_2$, respectively (Fig. 6B, C). For the experiments, two transparent tubes (6 mm inner diameter) are filled with Milli-Q water and 10 mg of microparticles each. The tubes are located within the workspace using two plastic dome nuts (Fig. 6D). The microparticles of reduced iron are approximately located at the center of each tube. Thereon, the electromagnetic system is activated according to (3) as the magnetic pillars move toward opposed directions.

## Conclusions

We introduce non-uniform magnetic fields to explore the collective behavior of self-assembled magnetic pillars formed from microparticles of reduced iron. Our study utilizes an electromagnetic system composed of nine iron-cored coils, which provide field-shaping and spatially selective actuation capabilities within the workspace. We experimentally determine the dimensions of self-assembled magnetic pillars and investigate four different actuation modalities. Through experimental validation, we demonstrate that a precessing magnetic field describing a Lissajous curve can be used for in-plane manipulation of a millimeter-sized glass bead. Furthermore, we examine the performance of magnetic pillars within a fluid-filled tube, providing insights into their ability to withstand and move under varying fluid flow conditions. Lastly, we leverage the potential of the nine-coil electromagnetic system to achieve the independent actuation of two clusters of magnetic pillars. Our results open up new avenues for further exploration and applications of self-assembled magnetic pillars exposed to non-uniform and time-varying magnetic fields.

## Experimental section

*Material and fabrication details:* The magnetic pillars are made of microparticles of reduced iron (12310, Sigma-Aldrich, USA). The microparticles are immersed into a suspension (1% weight) of surfactant (Tween 80, Sigma Aldrich, USA) in Milli-Q water. The workspace containing microparticles and suspension is a box (base dimensions: 22 $\times$ 22 mm$^2$) fabricated by laser-cutting (Speedy 300, Trotec Laser, Austria) parts of polymethyl methacrylate (PMMA) sheets (3 mm thickness) and assembling them using transparent glue. For acquiring the magnetization curve, we prepare a sample of microparticles suspended (1:1 mass ratio) in polydimethylsiloxane (PDMS) (Sylgard 184, #101697, UK). The suspension is cured into a cylindrical mold (5 mm diameter and 0.6 mm height) at 70 °C for 2 h and placed in the vibrating-sample magnetometer. For object manipulation experiments, a 2 mm diameter glass bead (SFVEBO0020000, RGPBALLS S.R.L, Italy) is employed. In order to facilitate the tracking algorithm, the bead is conveniently stained green using a permanent marker.

*Instrumentation:* The mass of microparticles of reduced iron utilized in this study is measured on a high-precision scale (SECURA225D-1S, Sartorius Lab Instruments GmbH & Co. KG, Germany). The microparticles are visualized using a scanning electron microscope (JSM-7200F, JEOL, Japan) to reveal further morphological details. In addition, the magnetization curve of microparticles of reduced iron is obtained using a vibrating-sample magnetometer (GMW 3474-140, GMW Associates, USA). Two cameras (FLIR, Wilsonville, USA), positioned above and to the side of the workspace, are equipped with microscope objectives (Optem FUSION, Qioptiq Ltd., UK) to capture high-resolution images (2048 $\times$ 2048 pixels) with a pixel resolution of approximately 10 $\upmu$m. For experiments involving fluid flow, a syringe pump (NE-4000, New Era Pump Systems, USA) generates constant flow rates of Milli-Q water within a transparent tube.

*Simulations and image processing:* The simulations of the magnetic field and gradients are obtained from a current-to-field map (Ongaro et al., 2018a) that is implemented in MATLAB (version 2023a, MathWorks, USA) for visualization. The algorithms for tracking and computing geometric parameters (average height and diameter) of magnetic pillars are implemented in a custom script of MATLAB. *Please refer to the accompanying text for details* (S1 Text, Supplementary information).

## Supplementary Information

Below is the link to the electronic supplementary material.

Supplementary file 1. Geometry of magnetic pillars, tracking, and motion analysis (PDF 2073 KB)

Supplementary file 2. Dynamic collective behavior: Actuation modalities (MP4 209330 KB)

Supplementary file 3. Manipulation of a 2 mm glass bead (MP4 141859 KB)

Supplementary file 4. Magnetic pillars motion with fluid flow (MP4 72940 KB)

Supplementary file 5. Independent actuation of two clusters of magnetic pillars (MP4 92380 KB)

## Data Availability

The authors confirm that the data supporting the findings of this study are available within the article and its Supplementary information. Raw data that support the findings of this study are available from the corresponding author, upon reasonable request.
