# Supplementary material for: Non-uniform magnetic fields for collective behavior of self-assembled magnetic pillars
Source: Swarm Intell. 2024 Jul 7;19(4):317–32. doi: 10.1007/s11721-024-00240-z (PMC12602647; doi:10.1007/s11721-024-00240-z)
Supplement: Supplementary file 1 — Geometry of magnetic pillars, tracking, and motion analysis (PDF 2073 KB) [file 11721_2024_240_MOESM1_ESM.pdf]

Supplementary information for:
Non-uniform magnetic fields for collective behavior of self-assembled magnetic pillars

Juan J. Huaroto[1*], Franco N. Piñan Basualdo[1], Dionne Lisa Roos Ariëns[1], Sarthak Misra[1,2]

[1]Surgical Robotics Laboratory, Department of Biomechanical Engineering, University of Twente, 7522 NB, Enschede, The Netherlands.
[2]Surgical Robotics Laboratory, Department of Biomaterials and Biomedical Technology, University Medical Centre Groningen and University of Groningen, 9713 GZ, Groningen, The Netherlands.

*Corresponding author(s). E-mail(s): j.j.j.huarotosevilla@utwente.nl;
Contributing authors: f.n.pinanbasualdo@utwente.nl;
d.l.r.ariens@student.utwente.nl; s.misra@utwente.nl;

Geometry of magnetic pillars

We experimentally obtained average values for the height and base diameter of magnetic pillars. The magnetic pillars are generated using coil #9 of a nine-coil electromagnetic system, while videos are recorded from both top and side views (Figure S1A). All images are analyzed using a custom script in MATLAB (version 2023a, MathWorks, USA). Images obtained from the side view of the workspace are cropped to obtain an image of length ($L \in \mathbb{N}$) and height ($H \in \mathbb{N}$) in pixels. The cropped image is converted from color to grayscale, and subsequently binarized (Figures S1B① and S1B②). In the binary image, the position of a pixel along the horizontal axis is identified by the subscript ($j \in \mathbb{N} \cap [1, L]$). The number of black pixels (bp_j) along the jth column is used to determine the height of each column (h_j) as follows:

$$h_j = H - bp_j, \tag{1}$$

and is subsequently plotted as a black line (Figure S1B③). Furthermore, the mean root square value of h_j (represented as a red dashed line) is multiplied by a pixel

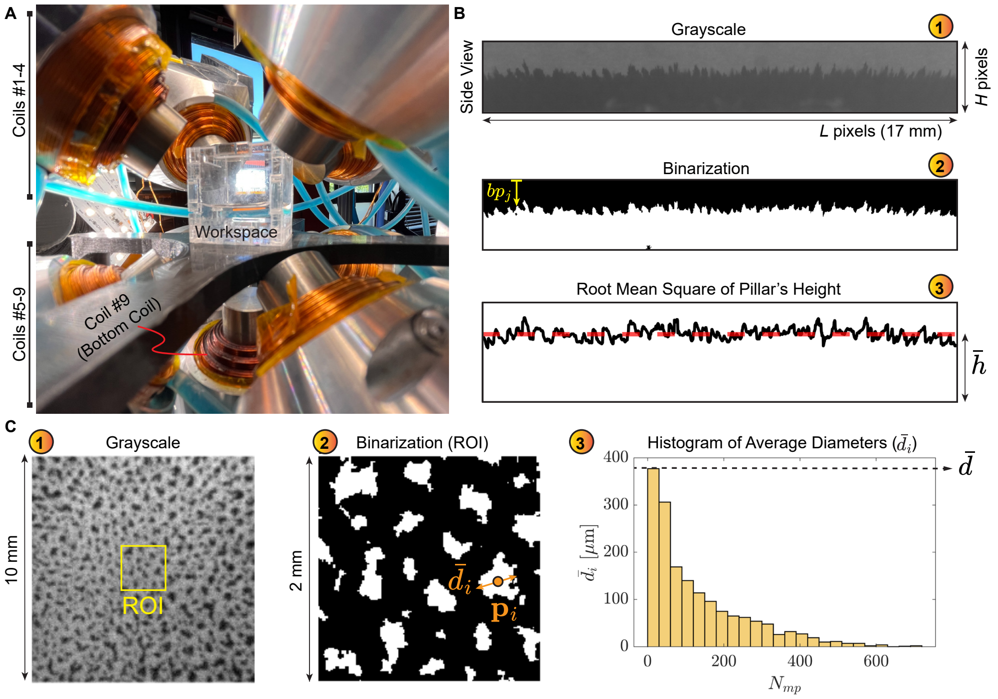

Fig. S1 (A) Nine-coil electromagnetic system used for experiments with magnetic pillars (Ongaro *et al.*, 2018). (B) Image processing to obtain the average height ($\bar{h}$) of magnetic pillars. The color images are processed as follows: ①, Crop and grayscale conversion; ②, Binarization showing the black pixels (bp_i) along the jth column; ③, The pillar's profile is identified and subsequently extracted as a dark line. The root mean square value of such a line is multiplied by a conversion factor to calculate $\bar{h}$. (C) The procedure to compute the average diameter ($\bar{d}$) of the base of magnetic pillars involves the following steps: ①, Crop and grayscale conversion; ②, Binarization to obtain the average diameter ($\bar{d}_i$) and position ($\mathbf{p}_i$) of each magnetic pillar; ③, Histogram showing the distribution of values of $\bar{d}_i$ according to the number of magnetic pillars (N_{mp}). The value of $\bar{d}$ corresponds to the maximum value depicted in the histogram.

to micrometers conversion parameter ($\lambda \in \mathbb{R}^+$) to calculate the average height of magnetic pillars ($\bar{h}$) in micrometers:

$$\bar{h} = \lambda \sqrt{\frac{1}{L} \sum_{j=1}^{L} h_j^2}. \tag{2}$$

In order to obtain the average diameter ($\bar{d}$) of magnetic pillars, we analyzed a cropped region measuring 10 × 10 mm^2 from the images captured by the top camera. Prior to binarizing the images, we enhanced their contrast using morphological top-hat filtering and contrast adjustment (Figure S1C①). Subsequently, the binarized images allowed us to identify the number of magnetic pillars ($N_{mp} \in \mathbb{N}$) and their centers of gravity. The ith magnetic pillar has a center of mass ($\mathbf{p}_i$) with coordinates along X and Y-axis

(xc_i and yc_i, respectively) (Figure S1C②). We obtained the projected area ($A_i \in \mathbb{R}^+$) of each magnetic pillar, which is used to calculate the diameter ($\bar{d}_i$) using the following equation:

$$\bar{d}_i = \lambda\sqrt{\frac{4A_i}{\pi}}. \tag{3}$$

Finally, the values of d_i are plotted in a histogram to obtain the average diameter ($\bar{d}$) of all magnetic pillars (Figure S1C③).

Tracking of magnetic pillars

The tracking of magnetic pillars is accomplished by calculating the center of mass of the collective. To achieve this, we analyze images obtained from the top view of the workspace using a custom MATLAB script (version 2023a, MathWorks, USA). Following the procedure to obtain the values of $\bar{d}$, we convert the color images to binary and subsequently compute the center of gravity of each pillar (p_i), which has coordinates along X and Y-axis (xc_i and yc_i, respectively). Moreover, we obtain the projected areas (A_i) of each magnetic pillar (Figure S1C②). Using these parameters, we determine the center of mass (p_{CM}) of the collective of pillars according to the following equation:

$$p_{CM} = \left[\frac{\sum_{i=1}^{N_{mp}} xc_i A_i}{\sum_{i=1}^{N_{mp}} A_i}, \frac{\sum_{i=1}^{N_{mp}} xc_i A_i}{\sum_{i=1}^{N_{mp}} A_i}\right]. \tag{4}$$

Motion analysis of the glass bead

In order to analyze the bead motion, the bead is located in three regions of the workspace (Figure S2A). A precessing magnetic field (10 mT amplitude, $I_9 = 2$ A, and $f_X = 1$ Hz) is used to actuate the magnetic pillars formed from 20 mg of reduced iron microparticles. The matrix $\left(\mathbb{M} = \begin{bmatrix} 2\,f_X & 0 \\ f_X & 0 \end{bmatrix}\right)$ encoding the bead motion toward the left direction contains the parameters to generate a magnetic field describing a Lissajous curve. We observe that the trajectory of the glass bead can be decomposed into translation (toward the left direction) and cyclic motion in the shape of a Lissajous curve (Figures S2B and C). During an actuation cycle, the magnetic pillars are oriented according to the magnetic field. The glass bead describes a cyclic motion in the shape of a Lissajous curve when interacting with the surrounding pillars. However, at the end of the actuation cycle, a net translation is caused by the non-reciprocal motion between the effective/recovery strokes (Figures S2B and C). For the motion analysis experiments, the translation is characterized using the moving average of the bead trajectory (Figure S2B). Figure S2C shows the cyclic motion described by the bead with respect to the instantaneous moving average position. The asymmetry observed in the Lissajous curves for regions (I and III) is caused by the non-uniform characteristics of the magnetic field within the workspace (S2 Video, Supplementary information: 00:42–00:49 s). This asymmetry gives place to a diagonal motion towards the center of

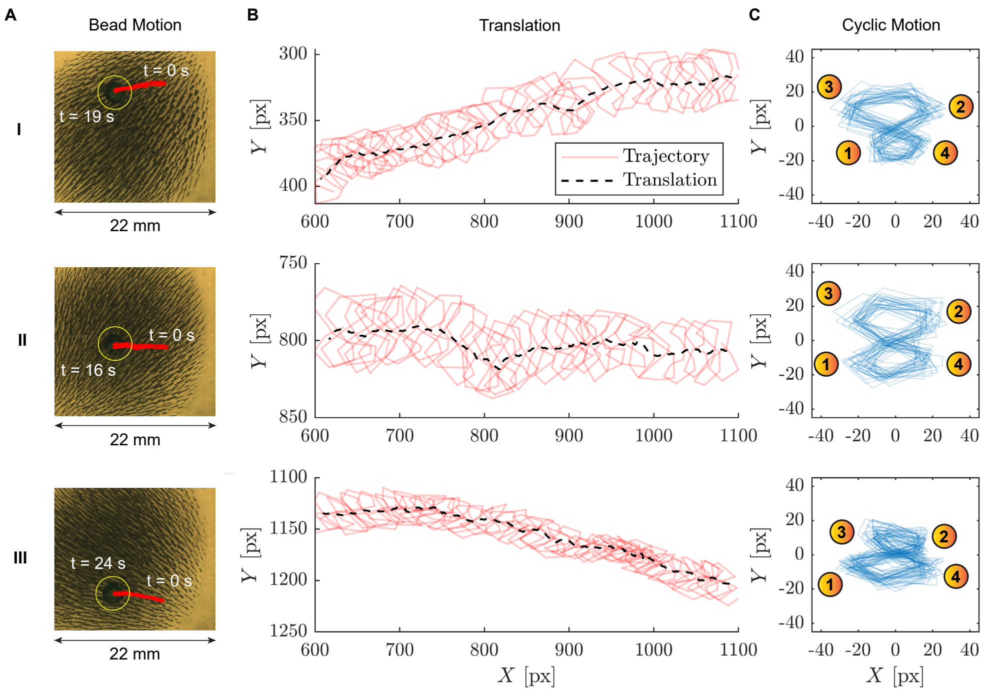

Fig. S2 Motion analysis of a 2 mm glass bead using 20 mg of reduced iron microparticles. (A) For the analysis, the glass bead (encircled in yellow) is initially located in three regions: I) "Top," II) "Center," and III) "Bottom" within the workspace. The bead trajectory is obtained experimentally and plotted as a red solid line. The trajectory is decomposed into translation and a cyclic motion in the shape of a Lissajous curve. (B) The translation (toward the left) of the bead is computed using a moving average filter. (C) The bead describes a Lissajous curve with respect to its instantaneous moving average position. The effective stroke is achieved within the intervals ②→③ and ④→①, whereas the recovery stroke is performed within ①→② and ③→④.

the workspace, as shown in Figure S2A and the manipulation experiments (S2 Video, Supplementary information: 00:58–01:34 s).

References

Ongaro F, Pane S, Scheggi S, *et al.* (2018) Design of an electromagnetic setup for independent three-dimensional control of pairs of identical and nonidentical microrobots. IEEE Transactions on Robotics 35(1):174–183
